# Cardio-Oncological Sequelae of Autologous Stem Cell Transplantation: Role of Clonal Hematopoiesis in Cardiovascular Risk

**DOI:** 10.1016/j.jacadv.2026.103279

**Published:** 2026-09-28

**Authors:** Zsófia Sándor, Zoltán V. Varga, Tamás G. Gergely, Laura Horváth, Tamás Masszi, Zsófia D. Drobni, José J. Fuster, Zoltán Pozsonyi

**Affiliations:** a Department of Internal Medicine and Haematology, Semmelweis University, Budapest, Hungary; b Department of Cardiology, Heart and Vascular Center, Semmelweis University, Budapest, Hungary; c Department of Pharmacology and Pharmacotherapy, Semmelweis University, Budapest, Hungary; d Center for Pharmacology and Drug Research & Development, Semmelweis University, Budapest, Hungary; e MTA-SE Momentum Cardio-Oncology and Cardioimmunology Research Group, Semmelweis University, Budapest, Hungary; f Centro Nacional de Investigaciones Cardiovasculares (CNIC), Madrid, Spain; g CIBER en Enfermedades Cardiovasculares (CIBER-CV), Madrid, Spain

**Keywords:** autologous hematopoietic stem cell transplantation, cardiovascular events, clonal hematopoiesis, onco-hematology, prevention, risk stratification

## Abstract

Autologous hematopoietic stem cell transplantation is a major milestone in modern oncohematological therapies; however, both in the peritransplant and post-transplant phase, the incidence of cardiovascular (CV) events is markedly higher than in the general population. The pathomechanism of CV complications is multifactorial, determined by complex interactions between patient-specific, transplant-related, and oncological risk factors. In recent years, clonal hematopoiesis has emerged as a clinically relevant determinant of adverse CV outcomes. Patients undergoing autologous hematopoietic stem cell transplantation represent a high-risk CV population due to cumulative cytotoxic exposure, treatment-driven clonal selection, and increasing long-term survival. These factors highlight the need for future development of clonal hematopoiesis–based risk stratification models that combine gene-specific alterations, clonal expansion magnitude, and mutational burden with conventional CV risk factors. Novel risk stratification approaches may enable tailored patient follow-up, individualized preventive interventions, and the early detection and prevention of CV complications in this sensitive patient population.

## Highlights

• Autologous hematopoietic stem cell transplantation is associated with increased cardiovascular (CV) risk.
• Clonal hematopoiesis (CH) driven by myeloid mutations contributes to CV risk.
• Autologous hematopoietic stem cell transplantation may expand pre-existing CH clones, potentially raising CV risk.
• CH-based risk stratification may improve CV surveillance and prevention.

## Autologous hematopoietic stem cell transplantation

Autologous hematopoietic stem cell transplantation (AHSCT) represents a revolutionary milestone in modern oncohematological therapy, fundamentally transforming the management of malignant hematologic disorders and selected nonhematologic diseases. In adults, AHSCT plays a major role in the treatment of multiple myeloma, Hodgkin and non-Hodgkin lymphomas, and, rarely, certain subtypes of acute myeloid leukemia (AML).^1^, ^2^, ^3^ Furthermore, it serves as an alternative therapeutic option for severe autoimmune diseases refractory to conventional treatments, including multiple sclerosis, systemic sclerosis, and Crohn disease.^1^^,^^4^, ^5^, ^6^

Advances in transplantation techniques, optimization of conditioning regimens, and improvements in supportive care have substantially enhanced survival following AHSCT.^7^^,^^8^ Consequently, an increasing number of transplant recipients now achieve long-term survival, including older individuals and patients with significant comorbidities who were previously considered suboptimal candidates for transplantation.^9^ According to the 2023 report of the European Society for Blood and Marrow Transplantation, 27,246 autologous transplants were performed, representing 57% of all hematopoietic stem cell transplantations.^10^ These demographic changes reflect the evolving landscape of AHSCT and highlight the growing relevance of age-related and treatment-associated long-term complications in transplant survivors.^11^

## Cardiovascular burden during AHSCT

Cardiovascular (CV) complications represent an increasingly recognized component of the overall morbidity associated with AHSCT.^12^, ^13^, ^14^, ^15^ Both during the acute peritransplant period (0-100 days) and in the late post-transplant phase (>100 days), the incidence of CV events is substantially higher than that observed in individuals of similar age from the general population.^16^, ^17^, ^18^, ^19^

The clinical relevance of this issue is highlighted by the fact that major adverse cardiovascular events (MACEs) may limit therapeutic success and adversely affect both the prognosis of the underlying hematologic disorder and the patient’s quality of life. Moreover, CV events during or following AHSCT are associated with a marked impairment of long-term survival and constitute an independent risk factor for both all-cause and nonrelapse mortality, particularly in the late post-transplant period.^12^^,^^20^^,^^21^ Early recognition of these complications is therefore essential and forms the basis of appropriate management and secondary prevention.^11^

## Mechanisms of CV injury in AHSCT

The underlying mechanisms of CV injury are multifactorial and primarily linked to the direct toxic effects of conditioning therapy, systemic inflammatory processes, endothelial dysfunction and, more recently, clonal hematopoiesis (CH), which is increasingly recognized as a potential contributor to CV injury after AHSCT.^12^^,^^13^^,^^22^

High-dose conditioning chemotherapy, a central component of the AHSCT protocol, is a major contributor to CV toxicity. The most commonly used regimens are high-dose melphalan (HD-Mel), BEAM (BCNU [carmustine], etoposide, cytarabine [Ara-C], melphalan), and, as an alternative, TEAM (thiotepa, etoposide, cytarabine [Ara-C], melphalan). Although these protocols are essential for effective myeloablation, they are associated with a spectrum of CV complications.^12^^,^^14^ Prior or cumulative exposure to anthracyclines, such as doxorubicin, together with high-dose alkylating agents including cyclophosphamide and melphalan, promotes excessive oxidative stress, mitochondrial injury, and activation of apoptotic signaling cascades within cardiomyocytes.^23^, ^24^, ^25^ These interconnected molecular insults predispose to both acute and progressive left ventricular systolic dysfunction, which may progress into overt dilated cardiomyopathy over time.^26^ HD-Mel, particularly, has been consistently associated with an increased incidence of supraventricular and atrial arrhythmias in the peritransplant setting.^27^, ^28^, ^29^ Furthermore, although the TEAM conditioning regimen demonstrates reduced pulmonary toxicity relative to BEAM, current evidence suggests a broadly comparable risk profile for CV complications.^30^^,^^31^

Although graft-versus-host disease is absent in AHSCT, conditioning and engraftment trigger cytokine release (such as interleukin [IL]-6, tumor necrosis factor-α), creating a systemic inflammatory milieu that exacerbates myocardial stress and promotes endothelial activation.^32^^,^^33^ Endothelial dysfunction—marked by impaired nitric oxide bioavailability, upregulated adhesion molecules, and a prothrombotic shift—contributes to early complications such as hypertension and thromboembolic events, as well as long-term atherosclerosis and atherosclerotic CV disease (ASCVD).^33^, ^34^, ^35^, ^36^, ^37^

Taken together, CV injury following AHSCT results from the complex interplay between cumulative exposure to cardiotoxic therapies, conditioning-related tissue injury, endothelial dysfunction, persistent inflammatory activation, and potentially CH-associated mechanisms, all of which contribute to both early and late CV complications.^12^^,^^14^

## Temporal patterns of CV complications

### Early (peritransplant) complications

Data indicate that during the early peritransplant period (0-100 days), new-onset arrhythmias are the most frequent CV complications, affecting 2% to 10% of adult recipients. The predominant forms include paroxysmal supraventricular tachycardia, atrial fibrillation, and atrial flutter, whereas ventricular arrhythmias are less common.^18^^,^^19^^,^^38^^,^^39^

In addition to arrhythmias, left ventricular dysfunction and acute heart failure may occur, especially in older patients, those with pre-existing CV comorbidities, or recipients receiving combination therapies. Pericardial complications, such as pericardial effusion, and, less commonly, ischemic events including myocardial ischemia or infarction, have also been reported, most often in association with HD-Mel.^27^^,^^40^^,^^41^

Myocarditis is a rare, but potentially life-threatening early complication.^12^^,^^42^^,^^43^ It is primarily associated with high-dose conditioning chemotherapy, particularly cyclophosphamide, which may induce direct myocardial toxicity through endothelial injury, oxidative stress, and inflammatory activation.^44^^,^^45^ The clinical spectrum is broad, ranging from asymptomatic left ventricular dysfunction to fulminant myocarditis complicated by acute heart failure, malignant arrhythmias, and cardiogenic shock.^12^^,^^45^^,^^46^

Thromboembolic events represent an important early complication, arising from the combined effects of endothelial injury, systemic inflammation, hypercoagulability, central venous catheterization, and prolonged immobilization. They most commonly manifest as deep vein thrombosis or pulmonary embolism, although, less common, arterial thrombotic events have also been reported following AHSCT.^47^, ^48^, ^49^

Endothelial dysfunction plays a pivotal role in the pathogenesis of several transplant-related complications. Although endothelial injury syndromes are less common after autologous than allogeneic HSCT, endothelial damage contributes to the development of transplant-associated thrombotic microangiopathy, sinusoidal obstruction syndrome, capillary leak syndrome, and long-term vascular dysfunction.^12^^,^^14^^,^^22^^,^^50^

### Late (post-transplant) complications

Long-term survivors of AHSCT constitute a population with elevated CV risk compared with the general population.^12^^,^^42^^,^^51^ The incidence of CV events, including arrhythmias, chronic heart failure, ischemic heart disease, stroke/transient ischemic attack, pericardial effusion, and both venous and arterial vascular diseases, is markedly higher.^52^, ^53^, ^54^ In addition, comorbid conditions such as hypertension, hyperlipidemia, diabetes mellitus, and metabolic syndrome indirectly increase CV risk.^11^^,^^13^^,^^18^, ^19^, ^20^ Global estimates of post-AHSCT CV events vary widely due to differences in the risk profiles of populations studied, with 10-year cumulative incidences ranging from 18% to 47%. Nevertheless, there is consensus that arrhythmias and heart failure are the most common long-term CV complications.^13^^,^^19^ Of particular importance, CV complications may emerge years after transplantation and often remain clinically silent during their early stages, manifesting as subclinical ventricular dysfunction, cardiomyopathy, conduction abnormalities, valvular heart disease, or pulmonary hypertension. This underscores the need for prolonged follow-up and lifelong CV surveillance to enable early detection and timely management, even in clinically stable AHSCT survivors.^12^^,^^14^^,^^19^^,^^55^

## New perspectives in oncohematology: clonal hematopoiesis as a CV risk amplifier

In the fields of cardio-oncology and oncohematology, increasing evidence supports a bidirectional relationship between CV and malignant diseases, extending beyond their shared risk factors.^56^, ^57^, ^58^, ^59^ Clinical and experimental studies in recent years have provided compelling evidence for this bidirectional interaction.^58^^,^^60^, ^61^, ^62^ On one hand, based on the reverse onco-cardiology approach, heart failure significantly increases the risk of developing malignancies.^63^ On the other hand, the association between cancer and the development of CV disease is also well recognized, as certain genes implicated in oncogenesis simultaneously promote the progression of both cardiac and vascular (atherosclerotic) diseases.^62^^,^^64^

Emerging clinical and translational evidence indicates that manifest ASCVD is independently associated with unfavorable oncological outcomes, particularly an increased propensity for metastatic disease.^57^^,^^65^ In a prospective cohort study involving over 21,000 individuals, incident ASCVD conferred a 75% increase in the risk of subsequent metastatic cancer diagnosis. Furthermore, the coexistence of ASCVD and metastatic malignancy was associated with significantly increased mortality.^57^ These observations reinforce the emerging paradigm that CV disease and cancer represent biologically interconnected conditions characterized by shared molecular, inflammatory, and immunological mechanisms that contribute to disease initiation, progression, and adverse clinical outcomes.^59^^,^^65^^,^^66^

CH, particularly CH of indeterminate potential (CHIP)-associated mutations, has recently emerged as a shared biological substrate linking aging, cancer, and CV disease. CH is an age-related condition characterized by the expansion of hematopoietic stem and progenitor cell clones carrying somatic mutations in genes recurrently mutated in myeloid neoplasms, in the absence of overt hematological malignancy or unexplained cytopenias. Although CH-associated myeloid driver mutations are relevant in a variety of hematological conditions, such as myeloid leukemias, myeloproliferative neoplasm, polycythemia vera, essential thrombocythemia, and primary myelofibrosis, CH is clinically relevant beyond hematology.^67^ A growing body of evidence links CH to increased risk of coronary artery disease, myocardial infarction, stroke, peripheral artery disease, systemic atherosclerosis, and prothrombotic or inflammatory CV phenotypes (Central Illustration).^68^, ^69^, ^70^, ^71^, ^72^, ^73^

**Central Illustration:**
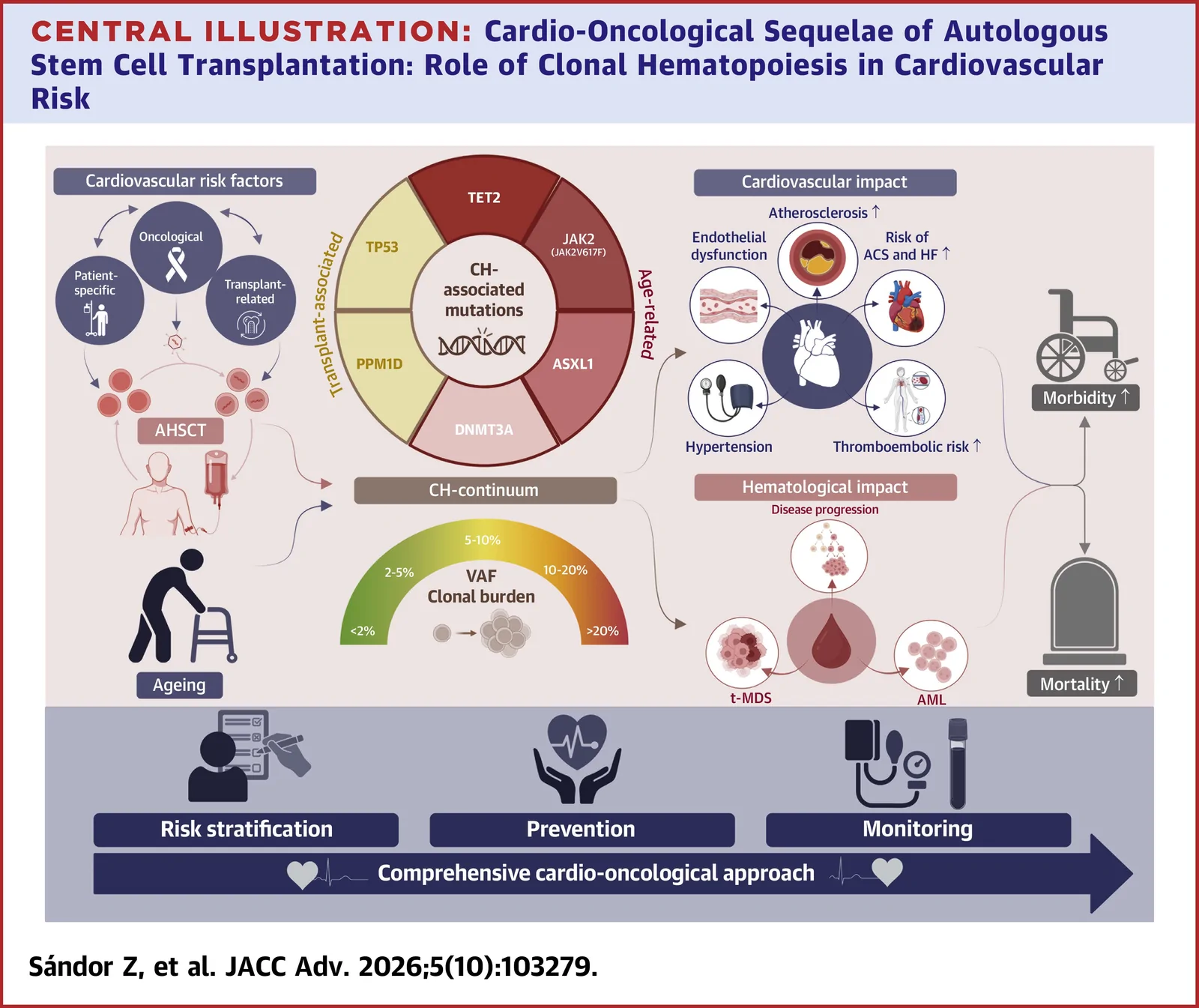
Cardio-Oncological Sequelae of Autologous Stem Cell Transplantation: Role of Clonal Hematopoiesis in Cardiovascular Risk The illustration summarizes the cardio-oncological sequelae of autologous hematopoietic stem cell transplantation, highlighting the emerging role of clonal hematopoiesis in cardiovascular risk. It integrates major clinical outcomes, including heart failure and acute coronary syndromes, with hematologic progression and mutation burden, emphasizing the need for comprehensive, risk-adapted management. The figure was created with BioRender https://BioRender.com. ACS = acute coronary syndrome; AHSCT = autologous hematopoietic stem cell transplantation; AML = acute myeloid leukemia; CH = clonal hematopoiesis; HF = heart failure; t-MDS = therapy-related myelodysplastic syndrome; VAF = variant allele frequency.

## The spectrum of clonal hematopoiesis: the continuum of gene-specific effects and clonal expansion

CH is an umbrella term describing the clonal expansion of hematopoietic stem cells harboring somatic mutations. One of the most well-characterized manifestations of CH is CHIP.^74^^,^^75^ CHIP is defined by the World Health Organization and the International Consensus Classification as the detection of 1 or more acquired somatic mutations in genes recurrently implicated in myeloid malignancies, present at a variant allele frequency (VAF) of at least 2%, in the absence of an established hematologic neoplasm or otherwise unexplained cytopenia.^76^, ^77^, ^78^ VAF represents the proportion of a specific genetic variant relative to all alleles at a given genomic locus.^79^^,^^80^ Although somatic variants may also be identified in genes not traditionally associated with myeloid cancers, their clinical implications remain less well understood and such mutations are not encompassed within the World Health Organization and International Consensus Classification diagnostic framework for CHIP. For example, CH with lymphoid driver genes has not been associated with increased coronary artery disease, in contrast to myeloid CHIP.^81^ Conversely, when CHIP-like mutations occur alongside persistent, unexplained cytopenias, the condition is classified as clonal cytopenia of undetermined significance (CCUS).^76^, ^77^, ^78^

Distinguishing CHIP from CCUS is of major clinical importance, as CCUS is associated with a substantially higher risk of subsequent myeloid malignant transformation and disease progression.^67^^,^^74^^,^^82^ In a cohort of 357 patients with CCUS, molecular risk stratification identified a high-risk subgroup with a 37.2% 2-year cumulative incidence of progression to myeloid neoplasms, further emphasizing the prognostic relevance of this distinction.^83^

The prevalence of CH increases markedly with age and is strongly influenced by the sensitivity of the sequencing method and the selected VAF threshold.^68^^,^^84^^,^^85^ The prevalence of CH is <1% in individuals younger than 40 years, 2% to 5% between 50 and 60 years of age, 10% to 15% in those older than 65 years, and reaches or exceeds 15% to 20% after the age of 70.^68^^,^^84^, ^85^, ^86^ Using ultrasensitive sequencing techniques, however, low-VAF clones can be detected in more than 40% to 50% of otherwise healthy older individuals.^84^^,^^86^^,^^87^ CCUS is considered rare in the general population, and its true prevalence remains unknown.^75^^,^^88^^,^^89^

Increasing clinical and pathological evidence suggests that CH should not be regarded as a uniform entity, but rather as a gene- and clone size–dependent condition with distinct clinical consequences.^90^, ^91^, ^92^ Based on this concept, the biological and clinical consequences of CH-associated mutations can be interpreted along 2 interrelated and integrated spectra. Accordingly, CH should be evaluated in the light of a continuum-based approach. One of these spectra is defined by the VAF, which reflects the extent of clonal expansion and the quantitative risk burden.^90^^,^^93^ The other is determined by the specific mutated genes, which shape the qualitative functional effects and directs the associated pathophysiological outcomes.^94^^,^^95^ These 2 spectra should not be considered independent dimensions; rather, they function as dynamically interacting dimensions that together determine the hematologic and CV phenotypic spectrum associated with CH.

CH is characterized by recurrent somatic mutations in myeloid driver genes. The most prevalent alterations affect epigenetic regulators (*DNMT3A, TET2, ASXL1, IDH1/2, KMT2A*), disrupting DNA methylation and chromatin state.^96^, ^97^, ^98^ DNA damage response genes (*TP53, PPM1D*) enable clonal selection under genotoxic stress. Mutations in spliceosome components (*SF3B1*, *SRSF2*, *U2AF1*) induce aberrant RNA splicing, while signaling and transcriptional regulators (*JAK2, CBL, BCOR*) perturb cytokine signaling and gene repression. Together, these lesions define the core molecular drivers of CH.^64^^,^^76^^,^^99^

The strongest CV associations are observed with *TET2* mutations, which are closely linked to coronary artery disease, ischemic stroke, and heart failure, consistent with the role of *TET2* in the epigenetic regulation of inflammatory gene expression.^100^, ^101^, ^102^ *TET2*-deficient monocytes and macrophages exhibit increased production of IL-1β and other proinflammatory cytokines, directly accelerating atherosclerosis progression and promoting endothelial dysfunction.^64^^,^^103^^,^^104^ Preclinical studies have demonstrated that *TET2* loss-of-function mutations drive CH through both intrinsic and extrinsic mechanisms. Aging hematopoietic stem cells intrinsically favor the expansion of *TET2*-mutant clones, whereas age-associated inflammatory cues, such as IL-1β and tumor necrosis factor-α, further promote IL-1R1-dependent proliferation, multilineage differentiation, and enhanced self-renewal of *TET2*+/− hematopoietic stem and progenitor cells. Particularly, genetic ablation of IL-1R1 or pharmacologic blockade of IL-1 signaling significantly attenuates *TET2*+/− clonal expansion, establishing the IL-1 pathway as a pivotal and therapeutically targetable driver of age-associated *TET2*+/− CH progression.^105^, ^106^, ^107^, ^108^

*ASXL1* and *JAK2* mutations primarily correlate with an increased risk of heart failure, atherosclerosis progression and thrombotic events.^72^^,^^109^^,^^110^ Notably, the JAK2V617F mutation is associated with a pronounced prothrombotic phenotype, potentially increasing both arterial and venous thrombosis risk by up to 12-fold and contributing to arrhythmogenesis.^64^^,^^99^^,^^111^

By contrast, *DNMT3A* mutations are generally associated with more moderate but broad-spectrum CV risk, particularly in the context of smaller clonal expansions.^99^^,^^112^

In 410,702 participants from the UK Biobank, the presence of CH (3.4%) was independently associated with increased risk of supraventricular, brady, and ventricular arrhythmias, with the strongest association for cardiac arrest. These relationships persisted after adjustment for coronary artery disease and heart failure and varied by arrhythmia subtype. Elevated risk was apparent across all CH driver genes, except for *DNMT3A*. Within this cohort, high-burden CH (VAF ≥10%) was also linked to greater myocardial fibrosis on cardiac magnetic resonance imaging (OR: 1.31).^113^

CH associated with *TP53* and *PPM1D* mutations represents a distinct CV risk profile that differs in several respects from the classical atherogenic CH gene effects. These mutations primarily impact key elements of DNA damage response and cell cycle regulation, often manifesting as therapy-associated clonal expansion, especially following chemotherapy or radiation.^87^^,^^114^ Although the hematologic progression risk of *TP53* and *PPM1D* mutations is well-established, growing clinical and epidemiological data also implicate their CV relevance.^72^^,^^114^, ^115^, ^116^ *TP53*- and *PPM1D*-mutant CH may define a distinct CV risk profile, particularly in patients exposed to cytotoxic chemotherapy or radiation. Because these genes are involved in DNA damage-response pathways, mutant clones may gain a selective advantage under treatment-related genotoxic stress.^87^^,^^114^ The CV relevance of these clones appears to encompass both atherosclerotic disease and nonatherosclerotic complications, including cancer therapy-related cardiotoxicity, heart failure, and increased CV mortality.^72^^,^^115^^,^^117^ In a study of 156 patients with therapy-related myelodysplastic syndrome (t-MDS), analyzed by targeted-capture sequencing of 295 cancer genes, *PPM1D* mutations were identified in one-fifth of cases and were strongly associated with prior exposure to DNA-damaging agents, particularly cisplatin.^118^ Mutant *PPM1D* confers a survival advantage under cisplatin-induced genotoxic stress, driving clonal expansion. Preclinical models support this mechanism, as cell lines harboring hyperactive *PPM1D* mutations outcompete wild-type cells following exposure to cytotoxic DNA-damaging agents, an effect primarily mediated by increased resistance to apoptosis. Furthermore, *PPM1D*-mutant cells do not display enhanced fitness during hematopoietic recovery after bone marrow transplantation. Together, these findings underscore the clinical significance of *PPM1D* mutations in CH, highlighting their impact on patient prognosis and the necessity of integrating mutation-driven risks into therapeutic management.^118^

The risk conferred by CH mutations is determined not only by the type of mutated gene but also by VAF, one of the strongest predictors of CV events. Several large cohort studies have demonstrated that CV risk is detectable even at low VAF levels (2% to 5%) and rises exponentially with progressive clonal expansion. Intermediate VAF ranges (5% to 10%) are associated with chronic low-grade inflammation and a measurable increase in CV risk. At higher VAFs (>10% to 15%), the incidence of MACE increases markedly, whereas very high clonal burdens (>20%) confer a further escalation of CV risk and adverse outcomes, indicating that clonal burden directly influences systemic inflammation intensity, vascular injury severity, and disease progression.^76^^,^^99^ However, although larger clone size is consistently associated with higher hematologic and CV risk, currently available evidence does not yet support universal VAF thresholds for clinical decision-making. VAF should be interpreted together with the mutated gene, number of mutations, prior cytotoxic exposure, blood counts, inflammatory status, and baseline CV risk. Consequently, the highest CV risk is observed in patients harboring high-risk gene mutations (*TET2*, *ASXL1*, *JAK2*) with large VAFs. In contrast, small *DNMT3A* clones typically confer milder, albeit not negligible, risk. Collectively, current observations support a stratified, continuum-based understanding of CH.^99^^,^^119^ Table 1 summarizes the continuum-based interpretation of CH.

**Table 1:** The Continuum-Based Interpretation of Clonal Hematopoiesis

		Gene		Dominant Cardiovascular Events
Cardiovascular risk	CH-Associated Mutations	Age-related classical atherogenic CH mutations	TET2	Acute coronary syndrome Coronary artery disease Heart failure (Cardiac decompensation) Stroke/TIA Peripheral artery disease Atherosclerosis progression Endothelial dysfunction
			JAK2 (JAK2V617F)	Acute coronary syndrome Coronary artery disease Stroke/TIA Peripheral artery disease Venous thromboembolic events
			ASXL1	Acute coronary syndrome Coronary artery disease Heart failure (Cardiac decompensation) Stroke/TIA
			DNMT3A	Acute coronary syndrome Coronary artery disease Heart failure (Cardiac decompensation) Stroke/TIA Atherosclerosis progression
		Transplantation-associated CH (TA-CH) mutations	TP53	Indirect cardiovascular risk Susceptibility to cardiotoxicity Heart failure development Increased cardiovascular mortality
			PPM1D	Increased risk of therapy-related myelodysplastic syndrome (t-MDS) and acute myeloid leukemia (AML) Worse long-term survival outcomes

Mutant Allele Fraction	<2%	2%–5%	5%-10%	10%-20%	>20%
Clonal burden	Minimal	Minor	Intermediate	Major	Predominant
Clinical associations	Often incidental	Minimal clinical impact	Classic CHIP Increased CV risk	Clinically relevant CHIP CV risk emerging Possible hematologic impact	Significantly increased CV risk and mortality; high hematologic progression risk
Variant allele frequency (VAF)					
Cardiovascular risk					

CHIP = clonal hematopoiesis of indeterminate potential; CV = cardiovascular; TIA = transient ischemic attack.

Recent advances in CH research have increasingly shifted from mutation-centered analyses toward systems-level, network-based, and data-driven approaches that capture the complex interplay between genetic, molecular, and clinical determinants of CH. Beyond traditional genomic studies, the integration of bioinformatics, multiomics profiling, single-cell technologies, network biology, and artificial intelligence is providing unprecedented insight into clonal evolution and its broader biological and clinical consequences.^120^, ^121^, ^122^, ^123^, ^124^, ^125^ This emerging framework positions CH not merely as a hematopoietic phenomenon but as a dynamic component of interconnected immune, metabolic, and CV networks, linking CH to the biology of aging and chronic disease.^126^ Ultimately, these advances may enable more precise risk stratification and support the development of personalized preventive and therapeutic strategies.^98^^,^^125^^,^^126^

## Clonal hematopoiesis in the context of AHSCT: from clonal evolution to clinical consequences

Over the past decade, emerging evidence has fundamentally reshaped our understanding of the biological and clinical relevance of CH in the transplant setting. Initially regarded primarily as a marker of age-associated clonal expansion, CH is now increasingly recognized as an active determinant of hematopoietic stress adaptation and a potential modifier of long-term outcomes following hematologic interventions.^68^^,^^127^^,^^128^

Among patients undergoing AHSCT, the prevalence of CH is higher than in the general population, ranging from 20% to 35% across different cohorts. This variability is influenced by several factors, including the underlying hematologic disease, patient age, prior exposure to cytotoxic therapy, as well as the sequencing methodology and the VAF threshold applied.^127^, ^128^, ^129^ In patients undergoing AHSCT for multiple myeloma or lymphoma, most studies applying the conventional CHIP definition (VAF ≥2%) have reported a prevalence of 25% to 35%.^12^^,^^127^^,^^128^ Reliable epidemiological data on CCUS in AHSCT recipients are currently lacking, and its true prevalence in this population remains to be determined.^75^^,^^128^^,^^129^

A growing body of evidence suggests that antineoplastic therapies, hematopoietic stressors, and regenerative processes do not merely alter clonal composition passively but rather exert selective evolutionary pressures on pre-existing mutant clones, promoting their persistence and expansion.^87^^,^^130^ In this context, AHSCT represents a particularly informative biological model, as hematopoietic reconstitution is derived from the patient’s own hematopoietic stem and progenitor cells. Consequently, pre-existing clonal alterations are reinfused with the autologous graft, where conditioning-induced hematopoietic depletion followed by intensive regenerative demand may create a selective environment that confers proliferative advantages to specific clones.^127^^,^^128^^,^^130^^,^^131^ As a result, the clinical implications of CH are likely to be more direct in the autologous setting than in allogeneic transplantation, where recipient hematopoiesis is largely replaced by donor-derived cells.^92^^,^^128^^,^^132^ Accordingly, the emergence and selection of CH in patients undergoing AHSCT are not determined solely by chronological aging. Prior exposure to chemotherapy, high-dose conditioning regimens, chronic inflammation associated with the underlying disease, and repeated cycles of hematopoietic regeneration may all contribute to the selective advantage of mutant clones.^87^^,^^94^^,^^130^^,^^133^ Clonal selection after cytotoxic therapy is gene-dependent and appears particularly relevant for CH driven by mutations in DNA damage-response genes.^87^ Accordingly, allele fractions of *TP53*- and PPM1D-mutant clones are often increased following AHSCT, reflecting the survival advantage of hematopoietic stem and progenitor cells harboring these mutations in the setting of chemotherapy-induced DNA damage.^127^^,^^134^^,^^135^ Consistent with this, analyses of lymphoma patients undergoing AHSCT have shown that pre-existing CH clones can persist and undergo post-transplant expansion, particularly when involving DNA damage response genes.^127^ These observations are further supported by a cohort of 81 patients with solid tumors or lymphoid malignancies undergoing AHSCT, in which CH was detected in 22% post-transplant, with a mean VAF of 10.7%. Most mutations were already present in the graft at lower VAFs, indicating a selective advantage of mutated hematopoietic stem cells during post-AHSCT hematopoietic reconstitution. Furthermore, the same study also observed that CH-associated mutations affecting DNA damage-response genes were associated with delayed neutrophil recovery, which also contributes to an increased long-term risk of CH-related complications.^136^

Concurrently, age-related CH mutations, including *TET2*, *JAK2*, *ASXL1*, and *DNMT3A*, may also be detected and undergo clonal expansion following AHSCT. However, the mechanisms driving their post-transplant dynamics remain incompletely understood and likely reflect a complex interplay between age-related CH, prior cytotoxic exposures, and transplantation-associated selective pressures.^69^^,^^87^^,^^137^^,^^138^

Clinically, persistent or progressive CH driven by myeloid mutations following AHSCT, particularly involving *TP53* or *PPM1D*, has been associated with an increased risk of t-MDS and therapy-related AML (t-AML), as well as worse long-term survival outcomes.^92^^,^^127^^,^^132^^,^^139^ Prospective studies have demonstrated that therapy-related myeloid neoplasms (t-MNs) frequently arise from pre-existing mutant hematopoietic clones.^131^^,^^140^ In patients undergoing AHSCT, CH detected at transplantation was associated with a significantly increased risk of subsequent t-MDS/t-AML, inferior long-term overall survival, and poorer CD34+ stem cell mobilization.^127^ Clonal evolution analyses further demonstrated that the leukemogenic mutations driving t-MDS/t-AML were often already detectable before transplantation, supporting the concept that these neoplasms frequently originate from the selection and expansion of pre-existing preleukemic clones.^127^^,^^140^, ^141^, ^142^

Although CH-associated mutations are not malignant in themselves, their premalignant potential points to the need for long-term monitoring of clonal dynamics and mutational spectra, especially in cases of high allele frequency, multiple concurrent mutations, or progressive clonal expansion.^91^^,^^143^

The clinical significance of CH, however, extends beyond clonal progression and the development of t-MNs. By the time of transplantation, most AHSCT recipients have accumulated substantial exposure to chemotherapy, radiotherapy, and other potentially cardiotoxic treatments, all of which independently contribute to long-term CV morbidity.^11^^,^^42^ Within this biologically vulnerable setting, CH driven by myeloid mutations may act in an additive or even synergistic manner to amplify CV injury. Because CH clones persist following AHSCT and frequently undergo further expansion during hematopoietic recovery, it is plausible that increasing clone size may parallel progressive enhancement of inflammatory activity and CV risk.^138^^,^^144^ This hypothesis is indirectly supported by clinical observations demonstrating that CH in patients undergoing AHSCT is associated not only with an increased incidence of t-MNs but also with higher nonrelapse mortality.^127^, ^128^, ^129^ Although the precise causes of excess nonrelapse mortality remain incompletely defined, CV disease represents a biologically plausible contributor, given the well-established association of CH with chronic inflammation, ASCVD, and CV mortality in nontransplant populations.^64^^,^^138^^,^^144^ In a cohort of 1,036 patients with multiple myeloma undergoing AHSCT, more than 20% harbored at least 1 CH-associated mutation before transplantation, with pre-existing CH being associated with a significantly increased 5-year incidence of CV disease, particularly in the presence of coexisting hypertension and dyslipidemia.^145^ These findings are of particular clinical relevance, given that CV complications remain a major cause of late morbidity and mortality among long-term survivors of AHSCT.^127^^,^^138^^,^^146^

Recent evidence further suggests that the impact of CH may extend to the earliest phases of the transplant process, including stem cell mobilization.^130^^,^^147^ In a single-center cohort comprising 776 patients undergoing evaluation for AHSCT, CH was frequently detected at the time of stem cell mobilization, even at relatively low VAF. Although the overall presence of CH was not independently associated with poor mobilization potential, mutations affecting DNA damage response pathways—particularly *PPM1D* and *TP53*—were enriched among patients who failed to achieve adequate peripheral blood stem cell collection for transplantation. Moreover, mobilization failure was associated with a higher incidence of t-MNs and inferior overall survival, although these adverse outcomes were not directly attributable to the presence of CH itself.^147^ Collectively, these findings suggest that specific molecular subtypes of CH may influence not only post-transplant clonal evolution and the risk of secondary myeloid malignancies but also the success of stem cell mobilization, a critical early step in the transplantation process.^130^^,^^147^ Particularly intriguing is the possibility that stem cell mobilization failure and CH share common biological underpinnings. Chronic inflammatory and metabolic alterations associated with impaired mobilization capacity are also well-established drivers of ASCVD.^64^^,^^144^ This raises the possibility that CH, diminished hematopoietic regenerative capacity, and adverse CV outcomes are linked through a shared pathophysiological axis shaped by inflammatory signaling pathways and stress-adapted hematopoiesis.^103^^,^^109^ Although the clinical significance and causal nature of these associations remain incompletely understood, current evidence suggests that CH may influence the entire spectrum of transplantation biology—from stem cell mobilization and hematopoietic reconstitution to long-term hematologic and CV outcomes.^128^

These findings may establish CH at the time of AHSCT as a powerful, clinically actionable biomarker of adverse outcomes, identifying patients at an increased risk of therapy-related myeloid malignancy, mortality, and long-term CV complications. Together, they underscore the prognostic impact of pre-existing CH and cardiometabolic risk factors in shaping both hematologic and CV outcomes after AHSCT.^127^^,^^132^^,^^139^^,^^145^

## International clinical recommendations for the CV risk and disease management in AHSCT

Effective prevention and management of both short- and long-term potential MACEs in AHSCT recipients requires multidisciplinary approach and close collaboration between hematologists, oncologists, and cardiologists. Detailed CV assessment, comprehensive cardio-oncologic risk stratification, and close follow-up should constitute integral components of the patient’s therapeutic algorithm.^11^^,^^19^^,^^148^^,^^149^

The European Society of Cardiology (ESC) published, for the first time in 2022, dedicated cardio-oncology guidelines, which also address hematopoietic stem cell transplantation, followed by the American Heart Association’s professional statement in 2024. Both documents cover pretransplant CV evaluation, risk stratification, and long-term monitoring of survivors, emphasizing the importance of interdisciplinary collaboration and the need for individualized, risk-oriented surveillance protocols.^19^^,^^148^

Previously, no evidence-based published guidelines existed specifically for pre-AHSCT CV assessment and risk stratification. Current treatment protocols are primarily based on expert consensus.^150^ Nevertheless, comprehensive CV risk assessment is a critical element across all stages of AHSCT.^19^^,^^148^

Before transplantation, evaluating the patient’s CV risk is essential to determine transplant eligibility and ensure procedural safety. Preparation for transplantation requires optimization of CV status, which may involve enrollment in cardiology care and regular monitoring.^19^^,^^148^

During the transplant process, close monitoring of acute CV complications is essential, with particular attention to the most common events, including atrial fibrillation, atrial flutter, and heart failure. Early detection and appropriate management are crucial, as a substantial proportion of these complications initially present as asymptomatic or subclinical forms.^19^^,^^148^^,^^151^^,^^152^

In the post-transplant phase, attention shifts toward long-term survival and sustained CV health surveillance. Longitudinal follow-up is aimed at preserving CV integrity and optimizing overall quality of life. In parallel, emphasis should be placed on lifestyle modification, promotion of physical activity, and structured support for patients’ reintegration into everyday life.^3^^,^^11^^,^^19^^,^^54^^,^^148^^,^^153^^,^^154^

## Risk assessment and monitoring during AHSCT

In routine clinical practice, identifying AHSCT recipients at risk for CV complications can be challenging due to the variability and complexity of individual patient factors and conventional risk factors alone may not fully capture the susceptibility underlying late post-transplant complications.^12^^,^^19^

The recently developed CARE-BMT (Cardiovascular Risk Assessment in Blood and Marrow Transplant Survivors) risk score represent a significant advance in the structured and standardized assessment of CV risk among hematopoietic stem cell transplant recipients.^19^^,^^149^ The risk score was derived from a large transplant cohort and was externally validated. The American Heart Association recommends its use for CV risk stratification.^18^^,^^19^^,^^149^ This simple-to-calculate tool integrates multiple variables to stratify CV risk in adult AHSCT patients. CARE-BMT is currently the only clinical decision-support tool developed using pretransplant variables in adult AHSCT recipients, and it has shown strong predictive performance in both autologous and allogeneic transplant populations.

The CARE-BMT model integrates 4 domains—demographic characteristics, cancer-related exposures, CV comorbidities, and laboratory markers—to stratify patients into 3 distinct risk categories, with corresponding 5-year cumulative incidence rates of CV events of 4%, 10.3%, and 22.4%. Understanding stratified risk profiles may guide individualized surveillance approaches and tailored preventive interventions.^18^^,^^19^^,^^149^

However, the risk score provide limited guidance on advanced CV monitoring strategies and do not yet incorporate the emerging CV risk associated with CH, which may contribute to the under-recognition of subclinical CV disease.

The 2022 ESC cardio-oncology recommendations classify patients into low- and high-risk groups for CV events. High-risk patients include those with prior mediastinal irradiation, exposure to alkylating agents, cumulative doxorubicin ≥250 mg/m^2^ or equivalent chemotherapy, conditioning protocols including total body irradiation or alkylating agents, and patients with multiple uncontrolled CV risk factors or established CV disease.^148^

Comprehensive CV assessment is recommended for both low- and high-risk groups, including physical examination, blood pressure measurement, lipid profiling, and glycosylated hemoglobin testing before HSCT, at 3 and 12 months post-transplant, and annually thereafter.

Electrocardiography is a class I recommendation (level of evidence C) at the same time points.^155^^,^^156^ Transthoracic echocardiography is advised pretransplant for low-risk patients, whereas high-risk patients should undergo additional echocardiography at 3 and 12 months post-transplant. Cancer therapy-related cardiac dysfunction is currently defined based on heart failure symptoms, reductions in left ventricular ejection fraction (LVEF) and/or relative changes in global longitudinal strain. Three-dimensional echocardiography is preferred for LVEF and volumetric assessment; if 3-dimensional is not feasible, the use of modified 2D Simpson’s method is recommended. Baseline LVEF and global longitudinal strain measurements are recommended in all patients to assess cancer therapy-related cardiac dysfunction risk and enable early detection of subclinical changes.^157^, ^158^, ^159^ Within the ESC risk stratification framework, reduced LVEF and established CV disease are considered high-risk features and relevant risk modifiers in the pretransplant setting, although they do not represent absolute contraindications to transplantation. Available clinical evidence indicates that a pretransplant LVEF ≥50% is associated with a favorable prognostic profile, whereas an LVEF of 40% to 49% acts as a major risk modifier, conferring a significantly increased risk of CV events and requiring intensified surveillance and targeted cardiologic optimization within structured clinical decision pathways. An LVEF <35% to 40% may be regarded as a relative contraindication and should prompt an individualized, multidisciplinary decision-making process integrating cardiac functional reserve and a comprehensive assessment of the transplant-specific risk-benefit balance.^38^^,^^158^^,^^160^^,^^161^

The use of cardiac biomarkers such as cardiac troponin I/T and natriuretic peptides (N-terminal pro–B-type natriuretic peptide) is less well established due to limited evidence in oncology. Their measurement is a class IIa recommendation before AHSCT in both risk groups, further assessment in low-risk patients is indicated only on the appearance of CV symptoms, whereas in high-risk patients its measurement is recommended at 3, 12 months, and annually.^148^^,^^152^^,^^162^, ^163^, ^164^

For selected high-risk patients, the ESC guidelines also recommend cardiopulmonary exercise testing as part of a comprehensive CV evaluation.^148^

Despite their important contribution to cardio-oncology practice, the ESC guidelines also have several limitations. Most recommendations are based on level C evidence, relying largely on expert consensus rather than prospective clinical validation, and the feasibility of the proposed surveillance strategies in routine clinical practice remains uncertain.^150^^,^^165^^,^^166^ In addition, similar to the CARE-BMT risk score, the ESC guidelines do not incorporate the emerging CV risk associated with CH.

## Beyond traditional risk factors: the role of CH in CV risk stratification in patients undergoing AHSCT

Current CV risk models rely primarily on traditional risk factors and inadequately capture the multifactorial mechanisms underlying CV complications. Consequently, CV risk in the AHSCT population may be underestimated.^12^^,^^19^^,^^146^ Moreover, these models lack validation in large transplant-specific cohorts, limiting their predictive accuracy. Omitting molecular and inflammatory contributors may particularly obscure risk in patients with otherwise favorable conventional profiles.^12^

Both the CARE-BMT risk score and the ESC cardio-oncology guidelines emphasize the importance of comprehensive CV risk assessment and long-term surveillance in patients undergoing AHSCT; however, neither framework currently incorporates CH as an independent CV risk factor.^19^^,^^148^^,^^149^

Although no validated CV risk prediction model currently incorporates CH, several prognostic models have recently been developed to estimate the risk of progression to myeloid malignancies in individuals with CH. These models may also provide indirect insights into CV risk, as emerging evidence suggests that higher-risk clonal profiles are associated with adverse CV outcomes.^74^^,^^167^

The CH Risk Score (CHRS) is a recently developed predictive model designed to estimate the risk of progression to myeloid malignancies based on variables including the affected gene, VAF, mutational burden, the presence of high-risk genetic alterations, and abnormalities in hematologic parameters.^74^ The CHRS represents an important advance by accounting for the considerable biological heterogeneity of CH. However, its applicability in the AHSCT setting remains limited, as the CHRS was developed to predict the risk of myeloid neoplasms rather than CV events. Although higher CHRS risk categories have been associated with an increased incidence of CV events, the model was developed and validated predominantly in nontransplanted populations and does not incorporate transplantation-specific factors, such as prior exposure to intensive cytotoxic therapy, reinfusion of an autologous graft harboring CH clones, and post-transplant clonal expansion. These factors may substantially influence both hematologic and CV outcomes, suggesting that the current CHRS alone is unlikely to provide optimal risk stratification for patients undergoing AHSCT.^168^^,^^169^

Like the CHRS, the Clonal Cytopenia Risk Score was designed to predict the risk of progression to myeloid neoplasia rather than CV outcomes. Developed specifically for patients with CCUS, it incorporates 3 readily assessable variables: ≥2 somatic mutations, platelet count <100 × 10^9^/L, and the presence of a spliceosome mutation.^83^ It effectively stratifies progression risk but was developed exclusively in CCUS cohorts and does not account for clonal burden, longitudinal clonal evolution, treatment-related exposures, or inflammatory factors, limiting its applicability to AHSCT recipients.

Similarly, the Myeloid Neoplasia Prediction Model was developed to estimate the risk of myeloid neoplasia rather than CV events. Derived from more than 450,000 UK Biobank participants, it is a multiparametric model integrating CH characteristics, VAF, blood count parameters, biochemical markers, and clinical variables, providing more accurate individualized prediction of myeloid neoplasia than simpler mutation-based scores.^170^ However, its utility in AHSCT recipients remains uncertain, as it has not been validated in transplant populations and does not fully capture longitudinal clonal evolution.

Taken together, although these models represent important advances in risk stratification for myeloid malignancies, no validated prediction model currently exists for CV risk assessment in individuals with CH, either in the general population or in patients undergoing AHSCT. This highlights an important gap in current CV risk stratification and underscores the need for novel prediction models incorporating molecular characteristics of CH. A growing body of evidence supports the potential value of this approach in patients undergoing AHSCT.^12^^,^^98^^,^^129^ The integration of CH status, mutational profile, and VAF dynamics with established clinical risk factors, CARE-BMT risk score, and cardio-oncology surveillance strategies may enable a more comprehensive evaluation of both hematologic and CV risk. Advances in next-generation sequencing (NGS) facilitate the characterization of clinically relevant molecular features, including clone size, mutational burden, and gene-specific alterations.^95^^,^^171^^,^^172^ Error-corrected ultradeep NGS enables highly sensitive detection and quantification of CH-associated somatic mutations, allowing identification of clonal variants at very low variant allele frequencies and improving characterization of CH.^173^ Although this approach substantially increases analytical sensitivity, its incremental clinical value for CV risk assessment has not yet been established.^174^ When considered alongside inflammatory and cardiac biomarkers, these parameters may further refine risk stratification and improve the identification of patients at increased risk for adverse hematologic outcomes and ASCVD.^144^^,^^175^ Although current evidence remains insufficient to support the routine implementation of CH testing or its incorporation into existing CV risk prediction models, CH-associated molecular alterations represent promising candidates for future transplant-specific risk assessment frameworks.^128^^,^^129^ A logical first step toward this goal would be the incorporation of routinely assessed CH-associated genes with the strongest evidence for adverse CV outcomes, including *DNMT3A*, *TET2*, *ASXL1*, and *JAK2*, together with transplantation-relevant genes involved in post-transplant clonal evolution and therapy-related clonal selection, particularly *PPM1D* and *TP53*. Future prospective studies are warranted to determine whether the integration of CH-related molecular markers into transplant-specific risk models can improve CV risk prediction, inform individualized surveillance strategies, and ultimately enhance long-term survivorship care following AHSCT.^73^^,^^128^^,^^129^

Figure 1 illustrates a conceptual framework for future CV risk stratification in patients undergoing AHSCT, integrating CH-associated molecular features with conventional CV risk factors.

**Figure 1:**
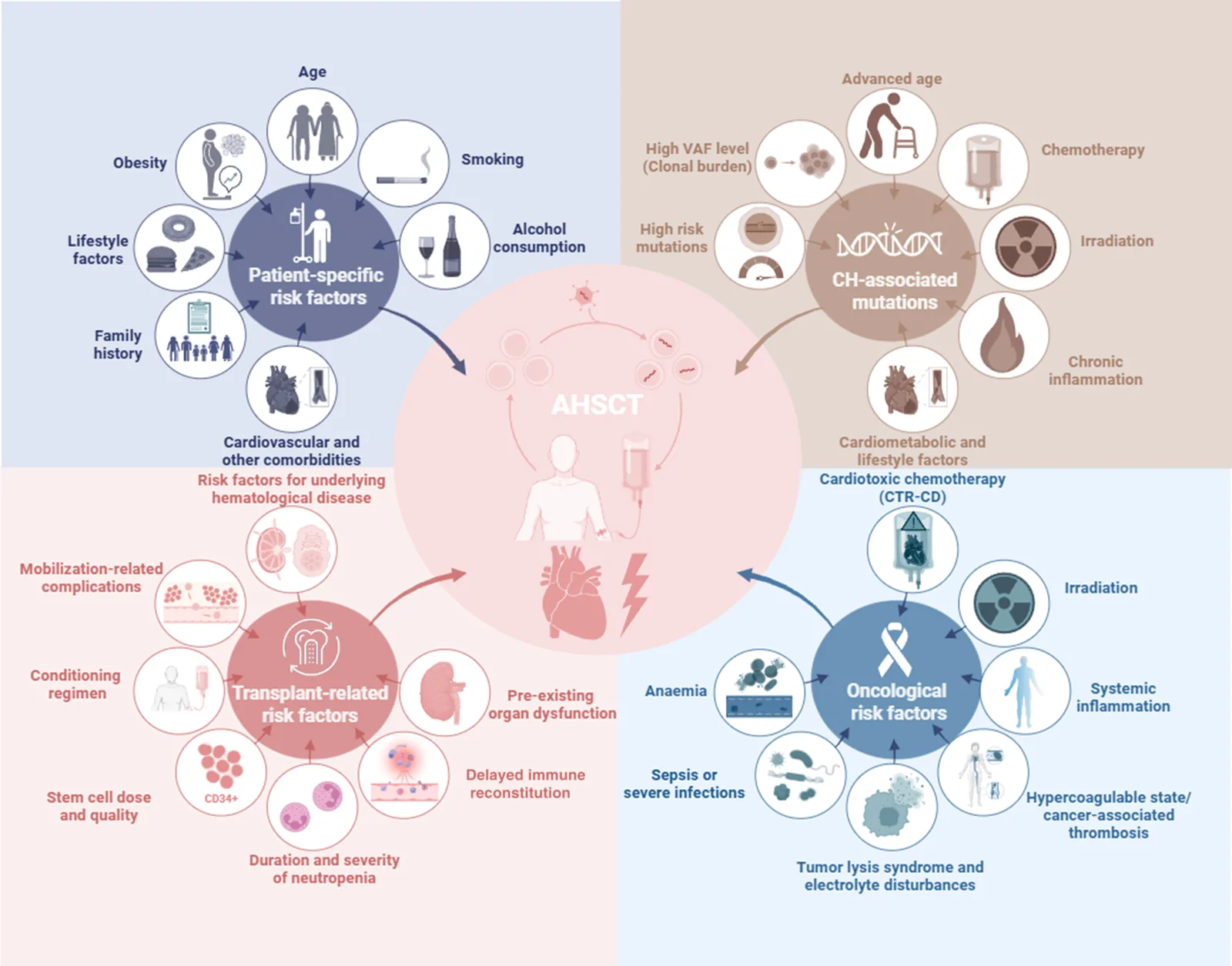
Determinants of Cardiovascular Risk in Autologous Hematopoietic Stem Cell Transplantation The figure illustrates the contribution of patient-specific, transplantation-related, and oncological factors, together with CH-associated mutations, to cardiovascular risk in patients undergoing autologous hematopoietic stem cell transplantation. The model highlights the multifactorial nature of risk and the role of genetic factors in refining stratification. The figure was created with BioRender (Created in https://BioRender.com). AHSCT = autologous hematopoietic stem cell transplantation; CTR-CD = cancer therapy-related cardiac dysfunction; CH = clonal hematopoiesis; VAF = variant allele frequency.

## CV prevention and management: current evidence and future directions

CV prevention in the context of AHSCT is based on the optimization of modifiable risk factors and lifestyle interventions, alongside targeted pharmacologic therapies.^12^^,^^73^^,^^95^

Current evidence does not support the use of any specific drug therapy for CV complications arising during AHSCT that differs from standard management in the nontransplant population.^12^^,^^42^^,^^146^ The administration of angiotensin-converting enzyme inhibitors, angiotensin II receptor blockers, beta-blockers, and statins—tailored to the patient’s individual CV reserve and risk profile—can substantially reduce the risk of both early and late CV events.^11^^,^^19^^,^^148^

Atrial fibrillation, one of the most common complications, should be managed according to standard treatment principles, modified to account for transplant-specific factors. Thrombocytopenia and bleeding risk often outweigh the CHA_2_DS_2_-VASc-predicted stroke risk, particularly in patients with severely reduced platelet counts, making the timing and modality of anticoagulation a matter for individualized clinical judgment. Available data suggest that direct oral anticoagulants can be safely used in this setting, but therapy requires careful personalized risk-benefit assessment, considering drug interactions and the patient’s current hematologic status.^176^, ^177^, ^178^

In the AHSCT setting, therapeutic targeting of CH remains particularly challenging, as most candidate interventions have been evaluated primarily in nontransplanted populations or preclinical models.^73^^,^^95^^,^^129^ Although CH is emerging as a potential therapeutic target, the unique biological context of AHSCT—including high-dose conditioning, transient aplasia, and prolonged immune reconstitution—requires careful evaluation of the risks and benefits of any strategy aimed at modulating clonal expansion.^12^ To date, no CH-directed therapy has been prospectively validated in patients undergoing AHSCT. Moreover, interventions affecting hematopoietic stem cell function may compromise graft recovery, prolong cytopenias, and increase the risk of infectious complications.^128^^,^^129^^,^^179^^,^^180^

For CH, no pharmacologic interventions or formal guidelines are currently available to eradicate it or prevent its progression or clinical consequences; therefore, it should be regarded as a high-risk condition rather than a directly treatable entity.^84^^,^^99^^,^^181^

Although recent post hoc analyses of CANTOS (Canakinumab Anti-inflammatory Thrombosis Outcomes Study), the LoDoCo2 (Low-Dose Colchicine 2) trial, and COLCOT (Colchicine CV Outcomes Trial) have provided preliminary evidence supporting the therapeutic modulation of CH, these findings require prospective validation before they can be translated into clinical practice.^182^, ^183^, ^184^ Consequently, current clinical management focuses primarily on structured monitoring, risk assessment, and targeted prevention, including optimization of conventional CV risk factors. Although several proposed clinical management approaches and risk-reduction strategies have recently been developed, their clinical utility remains to be prospectively validated, particularly in patients undergoing AHSCT.^67^ Hematologic follow-up should include regular complete blood counts every 6 to 12 months, whereas routine repeated NGS is not recommended.^185^^,^^186^ Repeated molecular evaluation should be considered only in the presence of clinical indications such as new cytopenia, or co-occurrence of multiple high-risk myeloid driver mutations.^97^^,^^119^^,^^187^

Figure 2 summarizes the core components of comprehensive cardio-oncological assessment during AHSCT.

**Figure 2:**
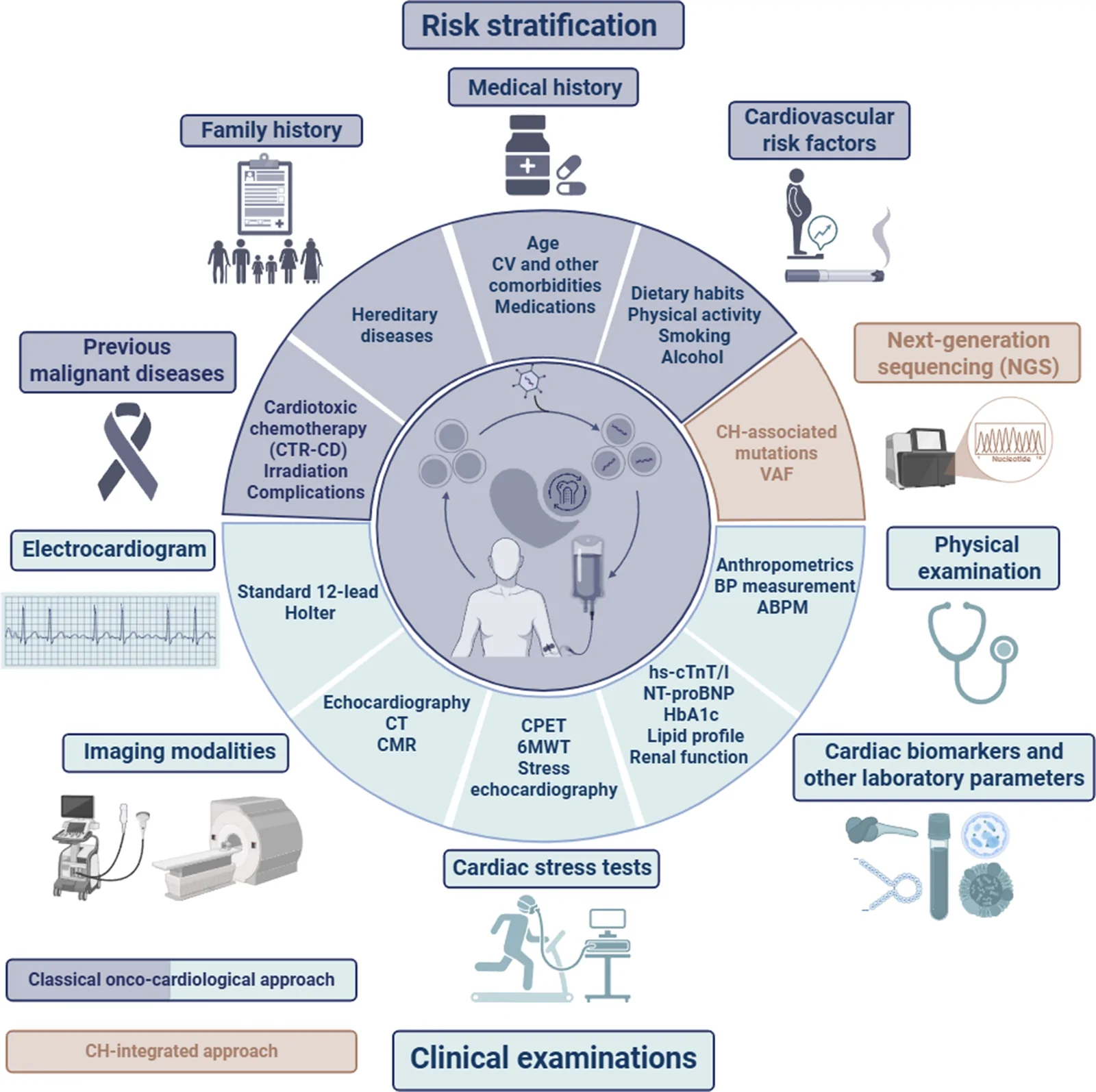
Cardio-Oncological Risk Assessment in Autologous Hematopoietic Stem Cell Transplantation: A Clonal Hematopoiesis–Integrated Approach The figure summarizes the core pillars of comprehensive cardio-oncological evaluation in patients undergoing autologous hematopoietic stem cell transplantation, incorporating CH-related parameters. Multimodal imaging, biomarker assessment, and genomic profiling are integrated to improve risk stratification and longitudinal monitoring. The figure was created with BioRender https://BioRender.com. ABPM = ambulatory blood pressure monitoring; BP = blood pressure; CMR = cardiac magnetic resonance; CT = computed tomography; CV = cardiovascular; CPET = cardiopulmonary exercise testing; HbA_1c_ = glycosylated hemoglobin; hs-cTnT/I = high-sensitivity cardiac troponin T/I; NT-proBNP = N-terminal pro–B-type natriuretic peptide; 6MWT = 6-minute walk test; other abbreviations as in Figure 1.

Current evidence suggests that patients harboring CH-associated mutations represent a population at elevated CV risk and may therefore benefit from more intensive management of modifiable CV risk factors, including smoking cessation, optimal control of blood pressure, lipid and glycemic levels, weight management, and regular physical activity.^11^^,^^185^^,^^188^ Accordingly, optimization of modifiable CV risk factors according to current ASCVD prevention guidelines, including statin therapy when clinically indicated, should be considered in patients with CH.^73^^,^^138^^,^^189^^,^^190^ Although CH is not an independent indication for therapy, its presence may help refine individualized risk stratification. Emerging observational data suggest that statins may attenuate CH-associated inflammatory pathways and reduce CV event incidence, although definitive evidence from randomized trials remains absent.^191^ Conversely, routine low-dose aspirin for primary prevention is not supported in CHIP, as CV benefit has not been demonstrated and bleeding risk may be elevated; its use should be confined to established secondary prevention contexts.^192^ However, prospective studies are warranted to confirm these recommendations, determine whether CH-directed CV risk-reduction strategies provide incremental benefit beyond conventional risk factor optimization, and define their role in patients undergoing AHSCT.

Therapies used for myeloproliferative neoplasms and AML, including JAK2 inhibitors (ruxolitinib, fedratinib), hypomethylating agents (azacitidine, decitabine), and isocitrate dehydrogenase inhibitors (enasidenib, ivosidenib), are not currently established for CV disease prevention in patients with CH. Their potential CV effects remain uncertain, with limited clinical data and some emerging preclinical evidence suggesting possible benefit.^193^, ^194^, ^195^

Several mutation-specific therapeutic strategies have recently emerged as potential approaches for targeting CH.^193^^,^^196^

In *TET2*-mutant CH, high-dose ascorbate has attracted considerable interest because of its role as an essential cofactor for *TET* enzymes. Preclinical studies have demonstrated that ascorbate enhances *TET*-mediated DNA demethylation, partially restores the epigenetic abnormalities associated with *TET2* deficiency, and suppresses the self-renewal and leukemogenic potential of *TET2*-mutant hematopoietic stem cells.^95^^,^^197^^,^^198^ Similarly, vitamin A and retinoic acid were shown to directly enhance *TET2* activity, thereby promoting epigenetic homeostasis and suppressing the expansion of CH.^199^

Moreover, preclinical studies indicate that the NLRP3-IL-1β axis is a central mediator of the CV effects of *TET2*-mutant CHIP, linking *TET2*-deficient hematopoiesis to macrophage inflammasome activation, amplified vascular inflammation, and accelerated atherosclerosis.^64^^,^^103^^,^^104^^,^^197^ In addition to mediating downstream CV injury, IL-1 signaling may also participate in a feed-forward loop that favors expansion of *TET2*-mutant clones. Bone marrow transplantation and genetic mosaic mouse models have shown that IL-1 can act as an extrinsic driver of *TET2*-mutant hematopoietic stem and progenitor cell proliferation through IL-1R1-dependent mechanisms. Consistent with this model, genetic or pharmacologic inhibition of IL-1 signaling can blunt mutant clonal expansion, supporting the concept that IL-1-targeted strategies may have dual relevance in *TET2*-mutant CH by attenuating both inflammatory CV injury and clonal expansion during aging.^101^^,^^105^^,^^106^^,^^200^ Building on these mechanistic insights, several protective strategies are under investigation, including NLRP3 inflammasome inhibitors, IL-1β blockade (canakinumab, decoy receptor rilonacept, IL-1 receptor antagonist anakinra), and colchicine.^182^^,^^201^^,^^202^ These approaches have shown efficacy in preclinical models, particularly in *TET2*-mutated contexts, and have reduced CV events in broader populations. Support for targeting CH has also emerged from post hoc analyses of major CV outcome trials. Notably, CANTOS demonstrated that canakinumab more effectively reduced MACE in patients with *TET2*-driven CH, highlighting the translational potential of targeting IL-1-mediated inflammation in CH.^182^^,^^202^ Similarly, exploratory analyses of the LoDoCo2 and COLCOT trials suggested that prolonged colchicine treatment may limit clonal expansion, raising the possibility that sustained suppression of inflammation could modify the underlying biology of CH in addition to reducing CV risk.^183^^,^^184^ Together, these observations provide an important clinical foundation for the development of therapies designed to directly restrain clonal expansion. However, in the absence of further, prospective CH-specific human data, these approaches remain experimental.^183^^,^^184^^,^^194^^,^^203^

In *DNMT3A*-mutant CH, metformin has emerged as a potential therapeutic candidate.^204^, ^205^, ^206^ The altered metabolic state and increased mitochondrial activity of *DNMT3A*-mutant hematopoietic stem cells suggest that the persistence and expansion of these clones may, at least in part, depend on metabolic adaptations. In experimental models, metformin reduced the competitive advantage and clonal fitness of *DNMT3A*-mutant cells through activation of AMP-activated protein kinase signaling, inhibition of mitochondrial complex I, and modulation of inflammatory pathways.^204^, ^205^, ^206^ These findings support the concept that metabolic vulnerabilities of *DNMT3A*-mutant clones may be therapeutically exploitable.^207^^,^^208^ However, clinical evidence remains limited, and current data do not support the routine use of metformin as a CH-directed therapeutic strategy.^209^

*TP53*-mutant CH has attracted particular interest, with recent translational studies providing the first evidence that such clones may be susceptible to pharmacologic intervention.^95^^,^^210^^,^^211^ The CDK4/6 inhibitor trilaciclib was shown to attenuate chemotherapy-induced expansion of *TP53*-mutant clones in both preclinical models and human samples derived from randomized clinical trials, primarily by preserving hematopoietic stem cell quiescence and reducing the selective advantage of mutant cells.^210^ These findings are particularly relevant in the AHSCT setting, where high-dose chemotherapy imposes substantial selective pressure on the hematopoietic compartment and may promote the expansion of *TP53*-mutant clones.^212^ However, the potential application of CDK4/6 inhibitors in this context warrants caution. In addition to their well-recognized hematologic toxicities—including neutropenia, leukopenia, and thrombocytopenia—which may impair post-transplant hematopoietic recovery and increase susceptibility to infectious complications,^213^, ^214^, ^215^ growing evidence suggests that these agents may also confer CV risk.^216^^,^^217^ Reported adverse events include QT interval prolongation, atrial and ventricular arrhythmias, hypertension, venous thromboembolism, and, less commonly, left ventricular dysfunction and heart failure.^218^ The CV toxicity profile appears to vary among the individual agents: ribociclib has been consistently associated with an elevated risk of QT interval prolongation, whereas treatment with abemaciclib has been reported to be associated with a higher incidence of venous thromboembolic events.^216^^,^^218^^,^^219^

Consequently, although CDK4/6 inhibition represents a promising strategy for modulating *TP53*-mutant clonal dynamics, its potential use in transplant recipients would require careful evaluation of both hematologic and CV safety.^214^, ^215^, ^216^

Overall, mutation-specific therapeutic strategies may offer a new perspective on the targeted modulation of CH; however, their clinical applicability remains largely hypothetical.^74^^,^^87^ In the context of AHSCT, the evaluation of these approaches should focus on their effects on hematopoietic recovery, graft reconstitution, and the risk of infectious and hematologic complications.^129^^,^^220^ The available evidence is currently limited predominantly to preclinical and early translational studies; consequently, routine clinical implementation cannot be justified at present.^221^^,^^222^ Further prospective, transplantation-specific clinical studies are required to define the role of these interventions in clinical practice.^220^^,^^221^

Figure 3 summarizes established and potential CV prevention strategies for patients with CH-associated mutations.

**Figure 3:**
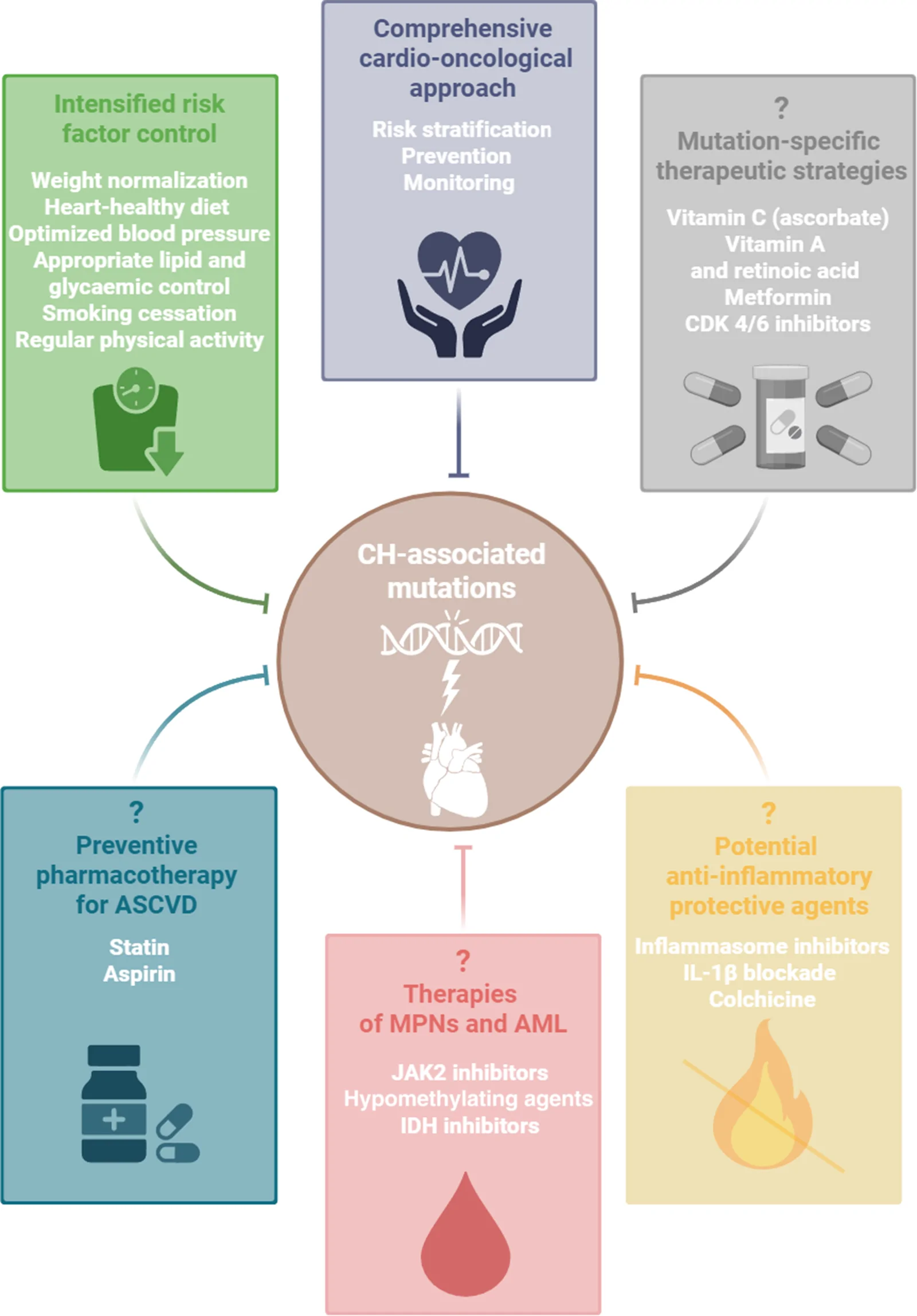
Cardiovascular Prevention and Management Strategies in Patients with Clonal Hematopoiesis–Associated Mutations The figure outlines the established and potential cardiovascular prevention and management strategies in patients with CH-associated mutations. It highlights the importance of cardiovascular risk factor management, targeted surveillance strategies, and emerging therapeutic opportunities aimed at mitigating CH-associated hematologic and cardiovascular complications. The figure was created with BioRender. AML = acute myeloid leukemia; ASCVD = atherosclerotic cardiovascular disease; CH = clonal hematopoiesis; CDK = cyclin-dependent kinase; IL-1β = interleukin 1β; IDH = isocitrate dehydrogenase; MPNs = myeloproliferative neoplasms.

## Conclusion: clinical implications and future directions

AHSCT provides an effective therapeutic option for various hematologic and an expanding number of nonhematologic indications, offering long-term survival even for high-risk, relapsed, or chemotherapy-refractory patients. However, CV complications during and after the procedure can significantly limit treatment success, negatively impacting both underlying disease prognosis and patient quality of life.

Although absolute mortality rates remain low, relative CV mortality is increased compared to the general population, underscoring the importance of early detection and appropriate management of these complications. Accordingly, risk stratification and targeted CV prevention play a key role, requiring multidisciplinary collaboration between hematologists, oncologists, and cardiologists.

The pathogenesis of CV complications is multifactorial, involving patient-specific risk factors, transplant-related treatments, and underlying oncologic disease. Conventional CV risk models alone do not adequately predict outcomes in AHSCT recipients, highlighting the need for integrated approaches. The emerging field of precision cardiohematology aims to integrate genetic, molecular, and clinical data to individualize CV risk assessment and management in patients undergoing AHSCT.

Future directions include the development of CH-based risk stratification models, incorporating gene-specific alterations, clonal burden (VAF), and mutational load alongside traditional risk factors and transplant-specific parameters. Integration of these models into clinical decision-making algorithms enables differentiated follow-up, tailored preventive strategies, and proactive management of CV complications.

Implementation in clinical practice requires prospective validation in multicenter cohorts to define the precise role of CH status in post-transplant standard care pathways. Further clinical studies, standardized protocols, and international consensus are required to harmonize therapeutic algorithms, thereby ensuring long-term survival, CV health, and overall quality of life in AHSCT recipients.

## Funding support and author disclosures

Dr Varga and this project received funding from the National Research, Development and Innovation Office and co-funded by the European Union NextGenerationEU under the umbrella of the Partnership Fostering a European Research Area for Health (ERA4Health) (GA N° 101095426 of the EU Horizon Europe Research and Innovation Programme). The work was supported by the Momentum Research Grant from the Hungarian Academy of Sciences (LP-2021–14 to Dr Varga). Dr Drobni and this paper were supported by the János Bolyai Research Scholarship of the Hungarian Academy of Sciences. All other authors have reported that they have no relationships relevant to the contents of this paper to disclose.
